# DeepAMR for predicting co-occurrent resistance of *Mycobacterium tuberculosis*

**DOI:** 10.1093/bioinformatics/btz067

**Published:** 2019-01-28

**Authors:** Yang Yang, Timothy M Walker, A Sarah Walker, Daniel J Wilson, Timothy E A Peto, Derrick W Crook, Farah Shamout, CRyPTIC Consortium, Irena Arandjelovic, Iñaki Comas, Maha R Farhat, Qian Gao, Vitali Sintchenko, Dick van Soolingen, Sarah Hoosdally, Ana L Gibertoni Cruz, Joshua Carter, Clara Grazian, Sarah G Earle, Samaneh Kouchaki, Yang Yang, Timothy M Walker, Philip W Fowler, David A Clifton, Zamin Iqbal, Martin Hunt, E Grace Smith, Priti Rathod, Lisa Jarrett, Daniela Matias, Daniela M Cirillo, Emanuele Borroni, Simone Battaglia, Arash Ghodousi, Andrea Spitaleri, Andrea Cabibbe, Sabira Tahseen, Kayzad Nilgiriwala, Sanchi Shah, Camilla Rodrigues, Priti Kambli, Utkarsha Surve, Rukhsar Khot, Stefan Niemann, Thomas Kohl, Matthias Merker, Harald Hoffmann, Nikolay Molodtsov, Sara Plesnik, Nazir Ismail, Guy Thwaites, Thuong Nguyen Thuy Thuong, Nhung Hoang Ngoc, Vijay Srinivasan, David Moore, David Jorge Coronel, Walter Solano, George F Gao, Guangxue He, Yanlin Zhao, Aijing Ma, Chunfa Liu, Baoli Zhu, Ian Laurenson, Pauline Claxton, Anastasia Koch, Robert Wilkinson, Ajit Lalvani, James Posey, James Jennifer Gardy, Jim Werngren, Nicholas Paton, Ruwen Jou, Mei-Hua Wu, Wan-Hsuan Lin, Lucilaine Ferrazoli, Rosangela Siqueira de Oliveira, São Paulo, Tingting Zhu, David A Clifton

**Affiliations:** 1 Institute of Biomedical Engineering, Department of Engineering Science, University of Oxford, Oxford, UK; 2 Oxford-Suzhou Centre for Advanced Research, Suzhou, China; 3 Nuffield Department of Medicine, University of Oxford, John Radcliffe Hospital Headley Way, Oxford, UK; 4 NIHR Oxford Biomedical Research Centre, John Radcliffe Hospital, Headley Way Headington, Oxford, UK; 5 Big Data Institute, Nuffield Department of Population Health, Li Ka Shing Centre for Health Information and Discovery, University of Oxford, Old Road Campus, Oxford, UK; 6 National Infection Service, Public Health England, Wellington House 133-155 Waterloo Road, London, UK

## Abstract

**Motivation:**

Resistance co-occurrence within first-line anti-tuberculosis (TB) drugs is a common phenomenon. Existing methods based on genetic data analysis of *Mycobacterium tuberculosis* (MTB) have been able to predict resistance of MTB to individual drugs, but have not considered the resistance co-occurrence and cannot capture latent structure of genomic data that corresponds to lineages.

**Results:**

We used a large cohort of TB patients from 16 countries across six continents where whole-genome sequences for each isolate and associated phenotype to anti-TB drugs were obtained using drug susceptibility testing recommended by the World Health Organization. We then proposed an end-to-end multi-task model with deep denoising auto-encoder (DeepAMR) for multiple drug classification and developed DeepAMR_cluster, a clustering variant based on DeepAMR, for learning clusters in latent space of the data. The results showed that DeepAMR outperformed baseline model and four machine learning models with mean AUROC from 94.4% to 98.7% for predicting resistance to four first-line drugs [i.e. isoniazid (INH), ethambutol (EMB), rifampicin (RIF), pyrazinamide (PZA)], multi-drug resistant TB (MDR-TB) and pan-susceptible TB (PANS-TB: MTB that is susceptible to all four first-line anti-TB drugs). In the case of INH, EMB, PZA and MDR-TB, DeepAMR achieved its best mean sensitivity of 94.3%, 91.5%, 87.3% and 96.3%, respectively. While in the case of RIF and PANS-TB, it generated 94.2% and 92.2% sensitivity, which were lower than baseline model by 0.7% and 1.9%, respectively. t-SNE visualization shows that DeepAMR_cluster captures lineage-related clusters in the latent space.

**Availability and implementation:**

The details of source code are provided at http://www.robots.ox.ac.uk/∼davidc/code.php.

**Supplementary information:**

Supplementary data are available at *Bioinformatics* online.

## 1 Introduction

Tuberculosis (TB) is one of the top 10 causes of death worldwide (WHO, 2018); and cases that are resistant to the main antibiotics that are normally used to treat the disease are increasing. The phenotypic drug susceptibility test (DST) that determines which antibiotics can cure particular TB infections is slow, labour-intensive and expensive. Given the potential for onward transmission of untreated TB, it is thus urgent to speed up the process that prescribes the best drug to individual patients in order to reduce the risk of the infection spreading. Whole-genome sequencing of *Mycobacterium tuberculosis* (MTB), the bacterium causing TB, provides a faster alternative for identifying drug-resistant TB (Quan *et al.*, 2018). A number of single nucleotide polymorphisms (SNPs) have been identified as associated with resistance to individual drugs. Traditionally, the MTB resistance to a specified drug is determined by any one of these resistance markers, i.e. single drug resistance determinants. However, because of the universal use of a common first-line treatment and resistance accumulation, resistance co-occurrence is quite common between the antibiotics, especially between the first-line drugs: isoniazid (INH), rifampicin (RIF), ethambutol (EMB) and pyrazinamide (PZA) (WHO, 2010). For example, the RIF-resistant MTB is often resistant to INH. Two types of resistant MTB are specifically monitored by the World Health Organization (WHO): () multi-drug resistant TB (MDR-TB), defined as MTB that is resistant to at least INH and RIF and (ii) extensively drug-resistant tuberculosis (XDR-TB) involves resistance to the two most powerful anti-TB drugs, INH and RIF, in addition to resistance to any of the fluoroquinolones (such as levofloxacin or moxifloxacin) and to at least one of the three injectable second-line drugs (amikacin, capreomycin or kanamycin). The SNP variants previously identified for single drugs are often jointly used for detecting M/XDR-TB. However, the evolution of poly-resistant MTB strains (isolates that are resistant to two or more drugs but do not meet the definition of MDR-TB) is a complex dynamic process that will have been influenced by interactions between genes and drug-resistant phenotype. For example, not all SNP variants are equally important for determining poly-resistance. Van Rie *et al.* (2001) used three codons (*rpoB*531, *rpoB*526 and *katG*315) to identify 90% of the MDR-TB cases in a cohort of 61 patients. Hazbón *et al.* (2006) studied 608 INH-susceptible and 403 INH-resistant MTB, and found the mutations in *katG*315 were more common in the MDR isolates, while the mutations in the *inhA* promoter were more common in INH mono-resistant isolates. Moreover, the presence of SNP variants in a resistant strain may be also related to susceptibility. Sintchenko *et al.* (1999) studied 36 MTB isolates in Australia and concluded that amino acid substitutions at *Asp*516 and *Ser*522 in the *rpoB* gene in RIF-resistant MTB predicted rifabutin susceptibility for MDR-TB. Walker *et al*. (2015) studied 23 candidate genes of 2099 MTB isolates, where 120 mutations were characterized as resistance determining and 772 as benign.

In this study, we propose a multi-task model with deep denoising auto-encoder (DeepAMR) to predict resistance of four first-line anti-TB drugs simultaneously and develop DeepAMR_AMR, a clustering variant based on the DeepAMR, to learn clusters in latent space of the data. Our data include 8388 isolates with phenotypic DST for these four drugs. We compared the proposed method to the resistant SNP association-based method (baseline model), random forest and support vector machine for single-label learning, multi-label K-nearest neighbours (MLKNN) and ensemble classification chains (ECC) for multi-label learning. An advantage of the proposed model over the examined machine learning models is that it integrated both non-linear dimension reduction and multi-label learning into an end-to-end model, so the high-level abstract of data and multi-label classification can be learned jointly. The results showed that the DeepAMR outperformed the baseline model and the other machine learning model in AUROC for all case and obtained the best sensitivity except for predicting RIF resistance and MDR-TB.

Meanwhile, visualization using t-distributed stochastic neighbour embedding (t-SNE) to the latent space generated by DeepAMR reveals that the clusters in latent space are associated to lineage.

## 2 Materials and methods

### 2.1 Specimen and laboratory phenotyping

Our dataset includes 8388 isolates with DST for all the four first-line drugs, which is a subset of >13 000 isolates that were tested with up to 11 drugs. For isolates in this study, the DST was performed on each drug through an initial screening of resistance in a liquid culture, then confirmed using Lowenstein Jensen methods. In our dataset, lineage of 3000 isolates are unknown, 2200, 1300 and 1200 isolates belong to lineages of European, CentralAsia and EastAsia, respectively, while relatively fewer isolates belong to lineages of WestAfrica1, WestAfrica2 and Animal. The lineage distribution of our data is shown in Supplement A.

### 2.2 DNA sequencing and pre-processing

The details of DNA sequencing are provided in Walker *et al.* (2015). Nucleotide bases were called using standard filters on sequencing and alignment quality, as well as the number of reads for each base. After filtering, the nucleotide bases at certain positions that could not be called with confidence were denoted as null calls and were not used in our analysis. We applied the same pre-processing method as described in Yang *et al.* (2017).

### 2.3 DeepAMR and DeepAMR_cluster

The resistance prediction for the MTB is essentially a multi-label learning problem, where each isolate is resistant to a subset of the examined drugs. The input is mutations for individual isolates, such that the presence of a SNP variable is denoted as one and zero otherwise. On the one hand, DeepAMR integrates deep denoising auto-encoder and multi-label classification into an end-to-end model, so the output is phenotypes of all examined drugs. Two objectives of DeepAMR are to minimize reconstruction error and classification error. On the other hand, DeepAMR_cluster integrates deep denoising auto-encoder and clustering into an end-to-end model, where two objectives are to minimize unsupervised clustering and reconstruction error. In DeepAMR_cluster, the augmented cluster layer referred to a Github project of ‘Keras_Deep_Clustering’ (https://github.com/Tony607/Keras_Deep_Clustering.) and the number of clusters was determined between 4 and 10 by maximizing adjusted rand index. The evaluation metrics for classification include sensitivity, specificity, F1 score and AUROC. The architecture of both DeepAMR and DeepAMR_cluster are provided in Supplement C. To ensure the robustness of our model, we performed hyperparameters selection and the selected hyperparameters were then used across all bootstrap experiments. The details of hyperparameters and implementation are listed in Supplement G.

### 2.4 MLKNN and ECC

Multi-label K-nearest neighbour (MLKNN) (Zhang and Zhou, 2007) is derived from traditional K-nearest neighbour algorithm. For each new instance, its K-nearest neighbours are firstly identified, and then its label are determined based on the label sets of these neighbouring instances using maximum *a posteriori* (MAP) principle. We applied internal cross-validation to search hyperparameters of MLKNN for every training dataset. A vanilla classifier chain method learns multiple binary classifiers linked along a chain, and each time extending the feature space by all previous labels in the chain. To address the difficulty in selecting an optimal order, ensemble classifier chain (ECC) (Read *et al.*, 2011) combines the predictions of different random orders and, moreover, uses a different sample of the training data to train each member of the ensemble. An advantage of ECC is that aggregating predicted relevance sets of the individual chains effectively improves prediction performance. We applied logistic regression models as base classifiers of ECC and tested the number of chains by 10, 20, 30, 40 and 50. As the number of chains increased, the prediction improvement increased at the scale of 0.1%. Thus, we selected 20 chains across all evaluation to balance trade-ff between computational efficiency and performance improvement.

### 2.5 Training and testing

In every experiment, 30% data were hold out for testing purpose. Table 1 reported average results of 100 bootstrap samples. All machine learning models tuned hyperparameters by an internal cross-validation on training dataset of every experiment except for ECC and DeepAMR. The optimal hyperparameters of ECC and DeepAMR were chosen by minimizing hamming loss and used for all bootstrap experiments. All results were reported on hold-out test dataset. In all models, the threshold for prediction scores were determined based on the optimal cutoff point on the receiving operating curve (ROC) using the whole training dataset. In the testing stage, the trained model was used to predict the labels of testing data, where the MDR-TB was determined by the joint presence of predicted resistance for INH and RIF, and PANS-TB was determined by predicted susceptibility for all of the four first-line drugs. For SVM and RF, the class weight for individual labels was ratio of resistant to susceptible isolates in the training set. The DA method does not require training, so it was directly applied to the testing set. During the training stage, the deep denoising auto-encoders of both DeepAMR and DeepAMR_cluster were pre-trained using unlabelled data, then the each model was fine-tuned. All Batch Normalisation and Dropout layers were only used in the training stage to avoid over-fitting. The training process was stopped when the validation loss stopped improving after 5 epochs.

**Table 1.** Comparing performance on F1 feature set for prediction of INH, EMB, RIF, PZA, MDR-TB as defined by WHO and PANS-TB

	Models						
Drugs		DA	SVM	RF	MLKNN	ECC	DeepAMR
INH	Sen	93.5±0.8	$92.6^{*}\pm 0.9$	$92.3^{*}\pm 1.0$	$80.9^{*}\pm 4.6$	$92.4^{*}\pm 0.9$	$94.3^{*}\pm 0.9$
	Spec	98.8±0.2	98.6±0.3	98.3±0.3	$97.4^{*}\pm 2.3$	$99.0^{*}\pm 0.2$	$95.7^{*}\pm 0.7$
	AUROC	96.2±0.4	$96.4^{*}\pm 0.9$	$96.7^{*}\pm 1.5$	$91.3^{*}\pm 1.1$	$95.8^{*}\pm 0.5$	$97.7^{*}\pm 0.4$
	F1	95.2±0.5	$94.5^{*}\pm 0.6$	$94.0^{*}\pm 0.6$	$86.4^{*}\pm 1.4$	$94.8^{*}\pm 0.5$	$92.1^{*}\pm 0.8$

EMB	Sen	86.8±1.5	$85.6^{*}\pm 1.8$	$84.7^{*}\pm 3.0$	$69.8^{*}\pm 8.9$	$79.8^{*}\pm 2.2$	$91.5^{*}\pm 1.6$
	Spec	93.9±0.4	$93.9^{*}\pm 0.5$	$94.0^{*}\pm 0.8$	$95.7^{*}\pm 1.5$	$96.3^{*}\pm 0.4$	$93.4^{*}\pm 0.6$
	AUROC	90.4±0.8	$92.1^{*}\pm 2.5$	$92.6^{*}\pm 3.4$	$88.2^{*}\pm 1.6$	$88.5^{*}\pm 1.0$	$96.8^{*}\pm 0.4$
	F1	76.5±1.5	$75.8^{*}\pm 1.5$	$75.6^{*}\pm 1.6$	$70.2^{*}\pm 3.2$	$78.2^{*}\pm 1.3$	$77.8^{*}\pm 1.4$

RIF	Sen	94.9±0.7	$93.2^{*}\pm 1.0$	$90.9^{*}\pm 1.5$	$83.0^{*}\pm 3.7$	$91.8^{*}\pm 1.0$	$94.2^{*}\pm 1.0$
	Spec	98.7±0.2	$98.9^{*}\pm 0.3$	$97.9^{*}\pm 0.4$	$96.4^{*}\pm 1.4$	$99.3^{*}\pm 0.2$	$95.8^{*}\pm 0.5$
	AUROC	96.8±0.4	$97.1^{*}\pm 1.1$	$96.4^{*}\pm 2.0$	$91.3^{*}\pm 0.8$	$95.7^{*}\pm 0.5$	$98.2^{*}\pm 0.3$
	F1	95.1±0.5	$94.6^{*}\pm 0.7$	$91.7^{*}\pm 1.0$	$84.9^{*}\pm 1.4$	$94.4^{*}\pm 0.6$	$90.1^{*}\pm 0.9$

PZA	Sen	47.9±2.4	$78.6^{*}\pm 2.4$	70.4±7.5	$62.1^{*}\pm 9.3$	$72.7^{*}\pm 2.7$	$87.3^{*}\pm 2.1$
	Spec	98.2±0.2	94.4±0.5	95.5±1.2	$96.1^{*}\pm 1.8$	$96.1^{*}\pm 0.5$	$90.9^{*}\pm 0.9$
	AUROC	73.0±1.2	$89.5^{*}\pm 3.1$	87.9±5.6	$84.1^{*}\pm 1.4$	$85.1^{*}\pm 1.2$	$94.4^{*}\pm 0.7$
	F1	59.5±2.1	$72.1^{*}\pm 1.7$	69.3±2.8	$65.3^{*}\pm 3.2$	$72.7^{*}\pm 1.7$	$69.1^{*}\pm 1.9$

MDR	Sen	85.7±1.6	$86.1^{*}\pm 1.9$	$85.7^{*}\pm 3.0$	$70.2^{*}\pm 8.4$	$81.0^{*}\pm 2.3$	$96.3^{*}\pm 0.8$
	Spec	94.0±0.4	94.1±0.5	$94.1^{*}\pm 0.8$	$95.9^{*}\pm 1.3$	$96.3^{*}\pm 0.4$	$95.0^{*}\pm 0.5$
	AUROC	89.9±0.9	$90.1^{*}\pm 1.0$	$89.9^{*}\pm 1.3$	$83.0^{*}\pm 3.6$	$88.6^{*}\pm 1.1$	$98.7^{*}\pm 0.3$
	F1	76.0±1.6	$76.5^{*}\pm 1.5$	$76.1^{*}\pm 1.6$	$70.8^{*}\pm 3.2$	$78.6^{*}\pm 1.3$	$89.5^{*}\pm 0.9$

PANS	Sen	94.1±0.7	$92.5^{*}\pm 0.9$	$90.5^{*}\pm 1.0$	$81.6^{*}\pm 3.2$	$90.7^{*}\pm 0.9$	$92.2^{*}\pm 0.9$
	Spec	97.9±0.3	$97.9^{*}\pm 0.4$	$98.1^{*}\pm 0.5$	$95.9^{*}\pm 2.0$	$99.1^{*}\pm 0.2$	$96.7^{*}\pm 0.6$
	AUROC	96.0±0.4	$95.2^{*}\pm 0.4$	$94.3^{*}\pm 0.5$	$88.8^{*}\pm 1.1$	$94.9^{*}\pm 0.4$	$97.2^{*}\pm 0.4$
	F1	94.8±0.4	$94.0^{*}\pm 0.5$	$93.0^{*}\pm 0.7$	$85.8^{*}\pm 1.3$	$94.2^{*}\pm 0.5$	$92.6^{*}\pm 0.6$

*Note*: Sensitivity (sens), specificity (spec), area under receiving operating characteristic (AUROC) and F1 score are reported as mean and SD across 100 bootstrap samples. The *P*-value of performance measurement of the examined classifier compared to DA was obtained by Wilcoxon signed-rank test. *represents a *P*-value <0.05.

### 2.6 Permutation feature importance

Permutation feature importance (https://docs.microsoft.com/en-us/azure/machine-learning/studio-module-reference/permutation-feature-importance) computes importance scores for each of the feature variables, where the importance measures are determined by computing the sensitivity variation of a model to random permutations of feature values. The importance score is defined here as the unscaled sensitivity reduction in performance after shuffling the feature values.

### 2.7 T-distributed stochastic neighbour embedding

**Fig. 1.**
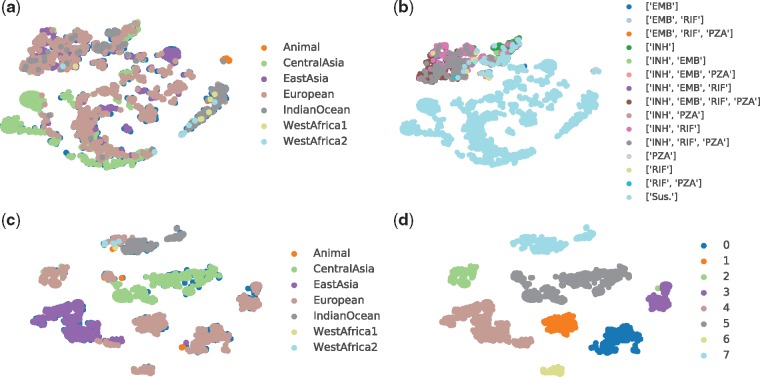
Illustration of latent structure using t-SNE: (**a**) lineage distribution resulted from DeepAMR; (**b**) phenotype distribution resulted from DeepAMR; (**c**) lineage distribution resulted from DeepAMR_cluster and (**d**) predicted clusters resulted from DeepAMR_cluster

T-distributed stochastic neighbour embedding (t-SNE) (Maaten, 2008) is a non-linear dimensionality reduction technique suitable for embedding high-dimensional data for visualization in a low-dimensional space of two or three dimensions. Assuming that close objects in high dimension are also closed in low-dimensional space, the t-SNE includes two main stages: (i) it constructs a probability distribution over pairs of high-dimensional objects so that similar objects have a high probability of being picked, while dissimilar points have a small probability of being picked and (ii) it defines a similar probability distribution over the points in the low-dimensional map, and minimizes Kullback–Leibler divergence between the two distributions with respect to the locations of the points in the map. For t-SNE in this article, the perplexity of 60, Barnes-Hut approximations and 800 steps were applied. In Figure 1(a) and (c), the samples are coloured by the label of lineage, while the samples are coloured by the DST results and predicted cluster labels in (b) and (d), respectively.

### 2.8 Iterative stratified cross-validation

To evaluate the proposed model and compare it with other methods, we split the dataset into training, validation and testing sets. We performed multi-label iterative stratified cross-validation (https://github.com/trent-b/iterative-stratification.) 100 times and report the average performance for testing dataset. It aims to ensure that the proportion of the two classes for every label are approximately the same in each fold. Given the imbalance of classes for each label and uneven label co-occurrence, e.g. the resistance of INH and RIF is highly related while the relationship between the resistance to other drugs is unknown, it cannot guarantee that the data of each fold are balanced. In each of the 100 bootstrap experiments, 30% testing data were hold out.

### 2.9 Related works

Machine learning algorithms are promising in the rapid prediction of mono-resistance to a single drug, although only a few have been specifically useful for analysing cross-resistance of MTB. Zhang *et al.* (2013) sequenced 161 MTB isolates with 94 MDR and 23 XDR cases and applied logistic regression to estimate the strength of association between the identified resistance-associated gene and intergenic regions and resistance to each drug. Farhat *et al.* (2016) adopted the random forest to predict both mono-resistance and MDR-TB cases. Resistance co-occurrence, i.e. cross-resistance, has been also addressed in other pathogens, e.g. viruses (Heider *et al.*, 2013; Riemenschneider *et al.*, 2016), where they addressed cross-resistance with multi-label approaches, ensemble classification chains (ECC). The ECC has been shown to outperform other multi-label models, including multi-label K-nearest neighbours (Cheng *et al.*, 2010).

We consider supervised non-linear Dimensionality Reduction (DR) in order to capture the discriminative latent space. Yu *et al.* (2005, 2006) proposed a Multi-label Informed latent Semantic Indexing (MLSI) model for supervised multi-label DR. It obtains the mapping matrix by solving an optimization problem, where the cost function is the trade-off between the reconstruction errors of both the input and output. Zhang and Zhou (2010) proposed dependence maximization (MDDM) for multi-label DR. The goal was to identify a lower-dimensional space by maximizing the Hilbert-Schmidt Independence Criterion between the original feature description and the labels in the subspace. Comparatively, the MLSI model considered label interaction based on matrix factorization of the output, while MDDM assumes the labels to be independent; both of them are solved by eigenvalue decomposition. Lin *et al*. (2014) proposed conditional principal label space dimensionality reduction by minimizing an upper bound of the Hamming loss, a trade-off between the prediction error and encoding error; the minimization was still implemented using singular value decomposition. Statically, Gönen *et al.* (2014) proposed a Bayesian Supervised Multi-label Learning (BSMLL) model coupled with DR and Multi-label Learning (MLL). The BSMLL model assumes linear projection and learns the projection model using variational approximation of posterior probability. These models are essentially matrix factorization. Although proposed as linear projection, they are able to extend to kernel projection. In comparison to DR and MLL successively, the model that jointly learns these two tasks could provide a more predictive subspace and improve prediction performance. Lacoste-Julien *et al.* (2009) proposed DiscLDA by extending the LDA model. DiscLDA and BSMLL are the same in assuming that the subspace is obtained by linear projection, although the former assumes that data follow the Dirichlet-Multinomial conjugate distribution while the latter assumes the Gamma-Gaussian conjugate distribution.

From the perspective of neural networks, Zhang and Zhou (2006) proposed backpropagation for multi-label learning with fully connected layers in order to minimize the global error function, i.e. the pair-wise difference of multiple labels. However, training a deep architecture was unsuccessful until unsupervised pre-training was proposed (Erhan *et al.*, 2009). Huang *et al.* (2013) proposed a Multi-task Deep Neural Network (MT-DNN) consisting of a five-layer model, where the three hidden layers were pre-trained as Gaussian Restricted Boltzmann Machine (RBM) and binary RBM by minimizing contrastive divergence. After pre-training, the MT-DNN was fine-tuned with labelled data by backpropagating the label assignment error. It is worth noting that the number of nodes in the three hidden layers was gradually increased. Qi *et al.* (2007) developed a correlative multi-label framework to learn correlation between video concepts. Huang *et al.* (2015) proposed multi-label conditional restricted Boltzmann machine for multi-label classification and label co-occurrence learning within a multi-task learning framework. Such model can be learned in a similar way as deep belief nets. Training RBM and auto-encoder works essentially to minimize the approximation of the log-likelihood of the generative model. Kiros and Szepesvári (2012) developed a hierarchical representation learning module with convolutional extraction and pooling, which can be stacked to form a deep model, followed by an auto-encoder with a single hidden layer, then exploited existing TagProp for image auto-annotation. Wicker *et al.* (2016) proposed multi-label classification using stacked auto-encoders (MANIAC), which applied stacked auto-encoders for non-linear label compression and then decomposed multi-label learning into multiple independent single-label problems using base classifiers. Among different variants of auto-encoders, DAE is more robust in its performance. It is at least as good as RBM when it is stacked into a deep supervised architecture (Vincent *et al.*, 2008). The stacked_DAE has been widely applied in various research areas for learning high-level representation of data, but there are a few works that are related to ours in the field of genetic data analysis. Zamparo and Zhang (2015) used stacked_DAE on cell images to reason cell biology or genetics, aiming to identify those cells which show interesting phenotypes and their sub-clusters. By using the genetic variation data (e.g. SNPs), similar to our study, Xie *et al.* (2016) applied a regression model of multilayer perceptron and stacked DAE with dropout to predict gene expression. Liang *et al.* (2015) adopted stacked DAE to cluster a combination of genotype, drug dosage and adverse drug event with the purpose of learning the relationship between the genetic polymorphism and adverse drug reactions. To the best of our knowledge, multi-task architecture integrated with stacked DAE has not yet been investigated for genome sequencing data.

## 3 Results

### 3.1 Phenotypes

**Fig. 2.**
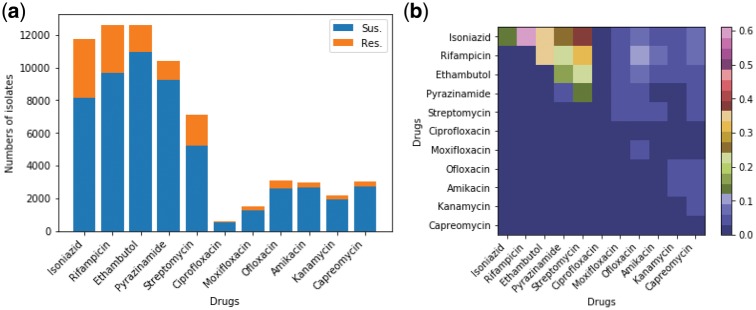
Overview of phenotype of the examined 13 403 MTB isolates. (**a**) Histogram showing the phenotype of the MTB isolates for each individual anti-TB drug obtained by the drug susceptibility test (up to 11 anti-TB drugs were tested for all isolates). For each drug, the isolates with missing phenotype were excluded. (**b**) Heatmap visualizing the proportion of pair-wise resistance co-occurrence (non-diagonal) and mono-resistance (diagonal) across anti-TB drugs. The non-diagonal elements correspond to poly-resistant isolates that were resistant to at least two anti-TB drugs. The co-occurrence matrix is symmetric so the upper right half of the graph shows all pair-wise co-occurrence cases

Figure 2 summarizes the phenotypes of the 13 403 MTB strains available for analysis. Figure 2(a) plots resistance and susceptibility from the phenotypic DST for isolates tested for individual drugs. Of 13 403 isolates, at least 80% were tested for INH, EMB, RIF and PZA, nearly 50% were tested for SM, less than a quarter for ofloxacin, capreomycin, amikacin, kanamicin and moxifloxacin, and only 0.5% for ciprofloxacin (the detailed numbers are provided in Supplement A). Most isolates were tested for EMB, where 10 892 (87%) were susceptible and 1558 (13%) were resistant. The 3393 INH-resistant isolates accounted for 30% of total isolates tested for INH. Among the first-line drugs, PZA had the fewest resistant isolates [1147 (11%)]. For the other second-line drugs, the percentage of resistant isolates ranged between 8% and 17%, although a smaller number of isolates had these drugs tested (Supplement A).

Figure 2(b) shows the pair-wise resistance co-occurrence (off-diagonal) and mono-resistance (diagonal). A mono-resistant isolate is defined as an MTB that was resistant to only one drug and susceptible to the other drugs. The numbers of the mono-resistant and two-drug resistant MTB in this map are normalized by the number of isolates that were resistant to at least any one of the examined drugs (*n* = 4038). It shows that the cross-resistance of INH and RIF is most frequent. The INH-streptomycin cross-resistance is ranked as the second most common cross-resistance. Less than 1% of isolates were mono-resistant to one drugs, except for INH where the proportion was 14%. The INH-EMB cross-resistance was similar to RIF-EMB accounting for 35% of resistant MTB. The proportion of INH-PZA cross-resistance was similar to that of RIF-PZA and EMB-streptomycin (22% to 24%). For the other drugs, there are far fewer cross-resistant and mono-resistant cases. Since there were few MTB strains with resistance co-occurrence for second-line drugs, we selected those MTB isolates with complete phenotypes for the four first-line drugs (*n* = 8388), for which all models were examined. The details of the cross-resistance are given in Table 2 of Supplement B. Meanwhile, Phi coefficients between first-line anti-TB drugs are shown in Table 3 of Supplement B, where positive Phi correlation across different drugs.

### 3.2 Direct association

The baseline method considered in this study aims to classify drug resistance based on the presence of any resistance determinant from a library of such determinants that has been assembled from the literature; we term this method ‘direct association’ (DA) using the resistance SNP catalog described in an existing study (Walker *et al.*, 2015), although we note that the method formally evaluated in this study also exploited ‘susceptible’ SNPs and had a third prediction class for novel mutations where the method classified samples as not being predictable. To classify resistance against a given drug in this study, we applied an ‘OR’ rule: if any of the mutations associated with a given drug in the library were present for a given isolate, then the isolate was labelled as being resistant to that drug, otherwise it was labelled as being susceptible. Moreover, an isolate with at least one resistance determinant for both INH and RIF was labelled as MDR-TB. On the other hand, an MTB without any resistance for all four first-line drugs was considered to be PANS-TB, which refers to isolate that is susceptible to all four first-line drugs. The DA method does not need to be trained because it uses a previously identified SNP catalogs, so it is robust to any issues with training set imbalance.

### 3.3 Comparison between baseline and machine learning models

In this section, totally six methods are evaluated, including the baseline model (DA), Random Forest (RF), Support Vector Machine (SVM), Multi-label K-nearest Neighbors (MLKNN) and Ensemble Classification Chain (ECC). The metrics for evaluating performance of classification include sensitivity, specificity, area under receiving operating characteristics (AUROC) and F1 scores (the harmonic mean of precision and recall).

The whole feature set (F1) includes 5919 SNPs found across all isolates, i.e. every isolate is represented by a vector with 5919 dimensions where every entry of the vector is binary. Table 1 lists the resulting metrics obtained by all examined models for the prediction of four first-line drugs, MDR-TB and PANS-TB using the whole feature set. For predicting INH, EMB and PZA resistance, DeepAMR achieved the highest mean sensitivity of 94.3%, 91.5% and 87.3%, respectively, and obtained the best AUROC of 97.7%, 96.8% and 94.4%, respectively. DA resulted in highest F1 score of 95.2% for INH and best specificity of 98.2% for PZA, followed by the SVM with sensitivity of 92.6%, 85.6% and 78.6%, with AUROC of 96.4%, 92.1% and 89.5%, respectively. RF performed similar to SVM in the case of INH and EMB but worse than the latter for PZA resistance prediction. ECC outperformed MLKNN and achieved the highest specificity of 99.0% and 96.3% for INH and EMB, and resulted the highest F1 score of 78.2% and 72.7% for EMB and PZA, respectively. Specially, DeepAMR improved the sensitivity and AUROC by 40% and 20% for PZA comparing to DA, respectively. With regards to RIF and PANS-TB, DA achieved its best sensitivity of 94.9% and 94.1%, and best F1 score of 95.1% and 94.8%, respectively. DeepAMR obtained the highest AUROC of 98.2% and 97.2%, respectively, followed by SVM with sensitivity of 93.2% and AUROC of 97.1% for RIF, and 92.5% and 95.2% for PANS-TB, respectively. For MDR-TB, DeepAMR produced the best sensitivity, AUROC and F1 score of 96.3%, 98.7% and 89.5%, respectively, while ECC generated the highest specificity of 96.3%. SVM had the second best sensitivity of 86.1% and AUROC of 90.1%. In aggregate, DeepAMR outperformed others approaches with the best AUROC for all drugs. It also had the highest sensitivity for all drugs except RIF and PAN-TB. The highest sensitivity for RIF was achieved using the DA method, but DeepAMR was ranked the best approach among the machine learning methods. In the case of MDR, DeepAMR achieved the best sensitivity, specificity and F1 score. After parameter optimization, SVM ranked the second best machine learning model, while MLKNN performed the worst. Ensemble classification chains (ECC) shows the best specificity in all cases except for PZA. The baseline method, DA, performed well for RIF and PANS by obtaining the best sensitivity and F1 score.

Moreover, all models were evaluated on a feature subsets (F2) where the previously identified SNPs were removed from the whole feature set, except for the DA, which was not able to predict resistance when the known SNPs were absent. For the sake of space, the results listed in Supplement D. DeepAMR obtained the best sensitivity except for PANS-TB, and it also had the best AUROC except for INH and RIF. Compared to results with feature set F1 in Table 1, the sensitivities of DeepAMR reduced to 76% for INH and PANS-TB, and it remained at least 80% for the other drugs. Meanwhile, the AUROC of DeepAMR with feature subset F2 were between 86% and 92% for all drugs. The F1 scores of all examined models were reduced by 5% to 20% for all drugs. Similar to results with feature F1, MLKNN and ECC still produced the best specificity. This analysis verifies that the machine learning models have potential for predicting resistance when the previously identified SNPs are absent. It can be explained that co-occurred mutation pattern associated to phenotype are used by machine learning models for resistance prediction.

### 3.4 Latent structure

In order to investigate relationship between lineage, phenotype and clusters of latent space, we considered both DeepAMR and its variant, DeepAMR_cluster. The former is a deep denoising auto-encoder augmented by multi-task classifiers, while the latter is augmented by a clustering layer. Please refer to Supplement C for the detailed structures of these two models. Figure 1 illustrates the latent structures at the bottleneck of the auto-encoder obtained by t-SNE for DeepAMR and DeepAMR_cluster, respectively. With regard to visualization for latent space of DeepAMR (as shown in Figure 1a and b), almost all lineages are scattered into different visual clusters except that the lineages of CentralAsia and IndianOceant dominant the bottom left cluster in green and the right cluster in dark grey, respectively. In terms of phenotype, resistant samples are more closed and separated from susceptible ones, although the subtypes of cross-resistance cannot be distinguished from each other (as shown in Figure 1b). It is observed that lineage and phenotype distribution of MBT are not related in latent space. On the other hand, the visualization for latent space of DeepAMR_cluster shows that several well-separated clusters corresponds to lineages, especially the lineages of EastAsia (purple dots) and CentralAsia (green dots) are distinct from others (as shown in Figure 1c). In Figure 1(d), the visual clusters are well-separated and consistent with the predicted clusters obtained by unsupervised clustering. Moreover, the clusters of 4, 5 and 7 correspond to the lineage of EastAsia, CentralAsia and IndianOcean, respectively. The rest clusters are also well-separated and correspond the same lineage of European as shown in Figure 1(c). The heat-map of confusion matrix between lineage and clusters obtained by the DeepAMR_cluster is provided in Supplement F. Comparing the latent structures resulted from DeepAMR with that of DeepAMR_cluster, it is implied that DeepAMR distorts the latent structure to better separate susceptible and resistant classes, yet DeepAMR_cluster captures the lineage-related clusters in latent space. Moreover, the visualization for the original input is also provided in Supplement E, where the t-SNE shows that the visual clusters are related to lineages, but the unsupervised clustering results shows that five of eight predicted clusters are mixed up.

### 3.5 Ranking of SNPs

**Fig. 3.**
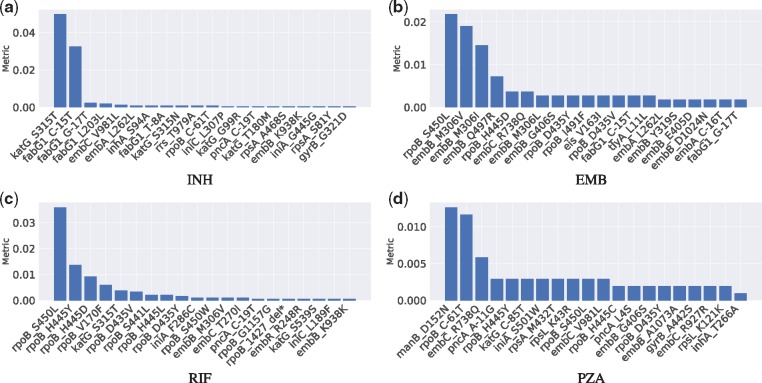
Ranked SNPs based on permutation feature importance resulting in positive metric with respect to INH, EMB, RIF and PZA, respectively

The SNPs ranking was obtained by DeepAMR based on permutation feature importance, where the metric was evaluated for every SNPs by permuting the order of one feature column at one time. The metric is sensitivity decrease after permutation. Figure 3 plots the ranked SNPs with respect to INH, EMB, RIF and PZA, respectively. For the sake of space, we only plotted top 20 SNPs with metric ¿0. On the one hand, the top three SNPs with respect to INH (as shown in Figure 3a), *katG S315T*, *fabG1 C-15T* and *fabG1 G-17T*, are previously identified INH-resistance determinants. On the other hand, shuffling the rest SNPs kept the sensitivity almost unchanged, where *inhA S94A* and *katG S315N* are associated to INH resistance, *embC V981L* and *rpoB C-61T* are lineage-related; *rpoB C-61T* is associated to Central Asian sub-lineage. Specially, *inhA S94A* and *fabG1 T-8A* are previously uncharacterized.

According to Figure 3(b), the top SNP, *rpoB S450L*, with respect to EMB is actually identified to be related to RIF resistance, while the next three SNPs, *embB M306I*, *embB M306V* and *embB Q497R*, are previously recognized to be associated to EMB resistance. Among the rest SNPs that changed the sensitivity minimally, *embB G406S* and *embB C-16T* are previously identified to be related to EMB resistance, *rpoB H445C*, *rpoB D435Y*, *rpoB I491F* and *rpoB D435V* are known to have a strong association with RIF, *fabG1 G-17T* is one of the most frequently seen INH mutation, while *embA C-16T* was considered as ‘benign’ (Walker *et al.* 2015).

In the case of RIF (as shown in Figure 3c), there are nine SNPs among top 11 SNPs known as RIF-resistance determinants; one of the other two is INH-resistance determinant, *katG S315T.* Among the rest SNPs, *rpoB 1427 delTGGCCC* and *embB M306V* are known as RIF and EMB resistance determinants, respectively, *embC T270I* was considered to define lineage.

With regard to PZA (as shown in Figure 3d), all top three SNPs, *manB D152N*, *rpoB C-61T* and *embC R738Q*, are associated with the sub-lineage of Central Asian. In the rest SNPs, *pncA A-11G* and *pncA L4S* are known to be strongly related with PZA resistance, *rpoB S450L*, *rpoB H445Y*, *rpoB H445C* and *rpoB D435Y* are known as RIF-resistance determinants, *embB G406S* is EMB-resistance determinant, *rpsL K43R* is known to be related to a second-line drug, streptomycin (SM). In addition, *iniA S501W* and *embC V981L* were identified as ‘benign’ and lineage-related, respectively.

## 4 Discussion

### 4.1 Discussion of results

The detailed description of hyperparameters optimization for all machine learning models is provided in Supplement H. For SVM, RF and MLKNN, the optimal hyperparameters were obtained with internal cross-validation in every bootstrap experiment. Hyperparameters of ECC and DeepAMR were chosen as those which provided the minimum hamming loss. The chosen hyperparameters are sub-optimal because there is trade-off between computational efficiency and performance improvement.

The results show that overall performance of two multi-label learning models, MLKNN and ECC, are worse than the other two single-learning models, SVM and RF. This can be explained that the classification errors in every labels accumulate jointly and then damage more to the two multi-label learning models. MLKNN determines the labels of a testing sample based on its neighbours using maximum a posterior principle, however it ignores the relationship between labels. ECC determines labels of a test sample by collectively considering multiple vanilla classification chains (CC), which sequentially treats each label as a binary classification problem. It overcomes the dependence to the order of labels in the vanilla CC, but has to go through all chains before making a decision for test samples and propagates errors through the chains. These two models are also limited in high dimensional sparse dataset because they were designed for low dimensional data. The DeepAMR takes advantage of high-level abstract of data, non-linear dimensionality reduction and then jointly learns the labels and reconstructs the data given the noise. Such nature improves the generalization capability of the DeepAMR. To sum up, comparing to the baseline model (DA), DeepAMR improved AUROC by 1.2% to 19% for all cases, and sensitivity by 1% to 39% for all except RIF and PANS-TB, whereas DA achieved 0.7% and 2% sensitivity higher than that of DeepAMR for RIF and PANS-TB, respectively. Compared with the second best machine learning model, DeepAMR increased AUROC by 1% to 8.5% for all cases, sensitivity by 1% to 9.7% for all except for PANS-TB; SVM gained 0.3% sensitivity higher than DeepAMR for PANS-TB.

In terms of evaluation metrics, both sensitivity and specificity are clinically important. In an imbalanced dataset with more negative samples (susceptible isolates), the specificity is generally higher than sensitivity. In the case when sensitivity (recall) is high but F1 score is low (e.g. EMB, PZA and MDR-TB), the precision is lower as F1 score is the harmonic mean of precision and recall. It means that positive samples that are falsely classified into negative group are more than negative samples falsely classified into positive group. It means that fewer of resistant isolates are treated as susceptible isolates yet relatively more susceptible ones are treated to be resistance. This is mainly because that the resistance co-occurrence pattern misled the classification of susceptible samples or there were errors in phenotype to four first-line drugs.

All models performed relatively worse for predicting EMB, PZA-resistance. This might be because: (i) the SNP patterns associated with resistance co-occurrence misled the classification for EMB and PZA resistance, (ii) the number of isolates that are resistant to EMB and PZA is relatively fewer than those to the other two drugs, (iii) genes outside of the considered 23 genes might be related to determine EMB and PZA-resistance and (iv) the resistance to EMB and PZA depends on a large number of resistance determinants who might be non-linearly related, which can be the reason why the DeepAMR improves the most for these two drugs, as well as for predicting MDR-TB, e.g. the sensitivity improves 5%, 40% and 10% for EMB, PZA and MDR-TB, respectively.

In all 13 403 isolates, only 8388 isolates have completed phenotype of first-line drugs to enable us to investigate cross-resistance in a supervised way. While the dataset is too small for optimal multi-class classification, multiple clusters were revealed in the latent space and learned by the proposed model via t-SNE visualization. The t-SNE attempts to preserve local structure: in other words, points that are close in the high-dimensional space remain close in the new low-dimensional space, which means that the ‘distance’ (affinities) between points in the resulting embedding space only reveals the neighbourhood relationship of points in the original high-dimensional manifold. The t-SNE is inefficient for high-dimensional dataset and its parameters are sensitive to the data. The resulting structure in embedding space corresponds to lineage and aligns to major clusters obtained by unsupervised clustering, which is basically consistent to clusters obtained by PCA in Supplement E.

According the ranked SNPs with regard to individual drugs, it is observed that INH and RIF resistance determinants dominant sensitivity variation for the classification of INH and RIF, respectively, while both RIF and EMB resistance determinants are highly important in sensitivity variation of EMB resistance classification. On the other hand, lineage associated SNPs contribute the most to sensitivity variation for PZA resistance classification, meanwhile RIF, EMB and SM resistance determinants also affect sensitivity variation of classifying PZA resistance. This is can be explained that PZA resistance is sensitive to the co-occurrence of resistance to RIF, EMB, SM and PZA.

### 4.2 Limitations of this study

The resistance co-occurrence is uneven between different drugs, e.g. INH and RIF resistance co-occurrence is highly frequent, while that of EMB and PZA is rarely seen. Assuming such prior knowledge is unknown, the objective function for every drug is equally weighted in the fine-tuning of DeepAMR during the training stage. We only considered cross-resistance between four first-line drugs but ignored that of second-line drugs because: (i) the inaccurate phenotyping for second-line drugs would introduce large error in the classification for first-line drugs and (ii) a small number of resistant isolates would result in over-fitting easily for such a complex model.

During the training stage, the class weight was computed for all machine learning classifiers, which might be different in the testing set, as it could result in increased covariance. Moreover, it is worth noting that the performance evaluation using sensitivity, specificity and AUROC might not be the best for evaluating multi-label learning, although we attempted to compare our model to the baseline method.

The visual clusters obtained by t-SNE are strongly influenced by the chosen parameters (such as perplexity, etc.) and could even appear in non-clustered data, and thus may be false findings. Another problem with t-SNE is that it does not preserve distances nor density but to some extent preserves nearest-neighbours. Moreover, t-SNE however suffers from limitations such as lack of an explicit out-of-sample extension (it cannot be used for fitting new sample), loss of information of the inter-cluster relationships), slow computation time, inability to meaningfully represent very large datasets. To overcome a shortcoming of t-SNE, kernel t-SNE as a parametric extension has been introduced in Gisbrecht *et al*. (2015). Kernel t-SNE preserves the flexibility of basic t-SNE but enables explicit out-of-sample extensions. More recently, Uniform Manifold Approximation and Projection (UMAP) has been proposed by McInnes and Healy (2018), claiming that it obtains similar results as t-SNE with a shorter run time and preserves more global data structure.

The permutation feature importance assumes that the variables are independent so it fails to recognize relationship between features. We acknowledge that permutation importance could introduce errors in SNP ranking. Several rare SNPs are ranked higher than other resistance determinants, even though all their effects to decrease sensitivity can be ignored. For example, in the case of INH, *embA L262L* ranked higher than *katG S315N*, yet there are three isolates contains the former and two of them are resistant to all four first-line drugs. Comparing with wrapper and filter methods for selecting features, a better ranking could be obtained by learning the importance of variables within the model.

## 5 Conclusion

Aiming to reduce dimensionality and classify multiple labels simultaneously, we proposed an end-to-end deep learning model, DeepAMR, by taking genomic data as inputs to classify drug resistance of MTB given resistance co-occurrence. The comparison results showed that DeepAMR outperformed the other models with best AUROC for all four first-line drugs, as well as MDR-TB and PANS-TB. It also achieved the highest sensitivity for all drugs except RIF. According to the visualization of the embedding space, DeepAMR distorts the latent structure of input data to better separate susceptible and resistant classes, while DeepAMR_cluster captures the lineage-related clusters in latent space of genetic data. In addition, it is observed that INH and RIF resistance determinants dominant sensitivity variation for the classification of INH and RIF, respectively, while cross-resistance pattern highly influences the prediction of EMB and PZA.

## Supplementary Material

btz067_Supplementary_Data
