# Supplementary material for: DeepAMR for predicting co-occurrent resistance of Mycobacterium tuberculosis
Source: Bioinformatics. 2019 Jan 28;35(18):3240–9. doi: 10.1093/bioinformatics/btz067 (PMC6748723; doi:10.1093/bioinformatics/btz067)
Supplement: btz067_Supplementary_Data [file btz067_supplementary_data.zip › btz067-suppl_data/main-supplement.pdf]

Supplement A

Table 1: Overview of phenotypes of isolates with respect to ant-TB drugs. (Tot. denotes total number of isolates. Sus. and Res. stand for the number of susceptible and resistant isolates, respectively, whose percentages are shown within the parentheses correspondingly. Untested means the number of isolates that were not tested against the drug.

DRUG	Sus. (%)	Res. (%)	Tot.
EMB	10829 (87%)	1558 (13%)	12387
RIF	9597 (78%)	2737 (22%)	12334
INH	8080 (70%)	3393 (30%)	11473
PZA	9267 (90%)	1147 (10%)	11414
SM	5105 (75%)	1729 (25%)	6834
OFX	2618 (85%)	458 (15%)	3076
CAP	2741 (90%)	315 (10%)	3056
AK	2690 (92%)	273 (8%)	2963
KAN	1925 (89%)	242 (11%)	2167
MOX	1249 (83%)	262 (17%)	1511
CIP	529 (87%)	77 (13%)	606

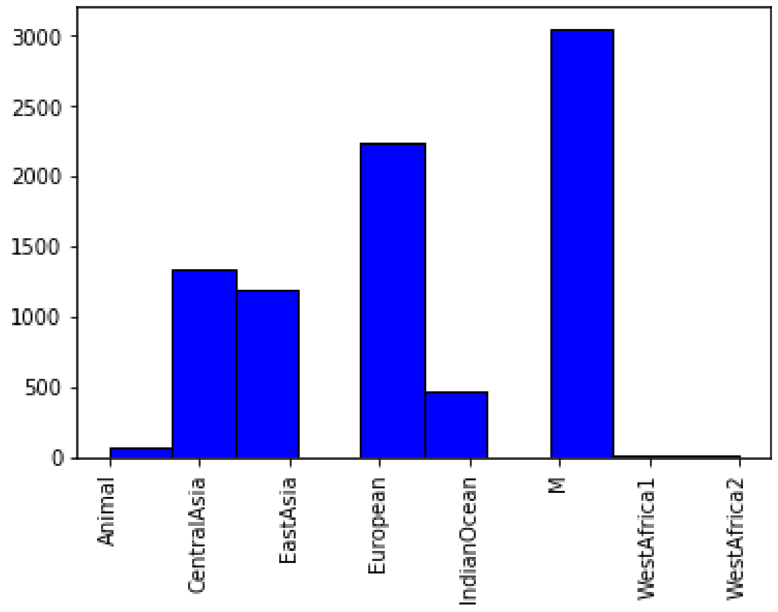

Figure 1: Lineage distribution of the dataset. “M” stands for missing values.

Supplement B

Table 2: Resistance co-occurrence summary for pair-wise drugs of the total 11 tested anti-TB drugs.

	INH	RIF	EMB	PZA	SM	CIP	MOX	OFX	AK	KAN	CAP
INH	552	2465	1410	988	1539	59	244	366	224	165	259
RIF	0	112	1446	930	1335	48	241	438	259	222	288
EMB	0	0	16	659	905	32	155	308	204	159	227
PZA	0	0	0	124	603	17	126	165	122	89	134
SM	0	0	0	0	115	9	130	228	171	83	202
CIP	0	0	0	0	0	2	23	22	1	2	1
MOX	0	0	0	0	0	0	2	225	61	55	63
OFX	0	0	0	0	0	0	0	1	117	132	133
AK	0	0	0	0	0	0	0	0	1	125	213
KAN	0	0	0	0	0	0	0	0	0	9	129
CAP	0	0	0	0	0	0	0	0	0	0	7

Table 3: Phi coefficients between first-line anti-TB drugs.

	INH	EMB	RIF	PZA
INH	1	0.59	0.76	0.48
EMB	0.59	1	0.69	0.55
RIF	0.76	0.69	1	0.55
PZA	0.48	0.55	0.55	1

Supplement C

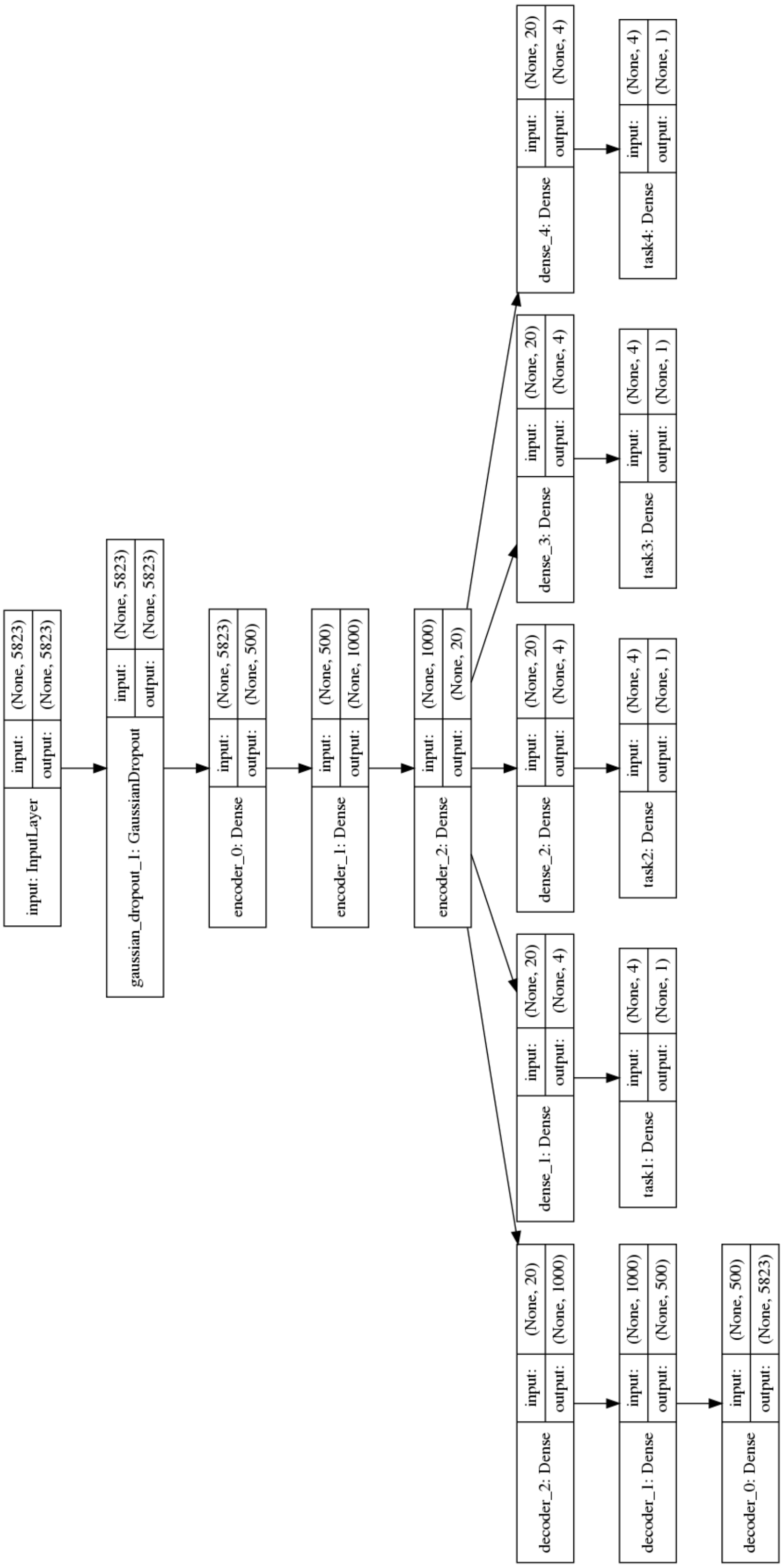

Figure 2: Architecture of deepAMR, where a deep denoising auto-encoder is augmented by multi-task classifiers at the most inner layer of the auto-encoder.

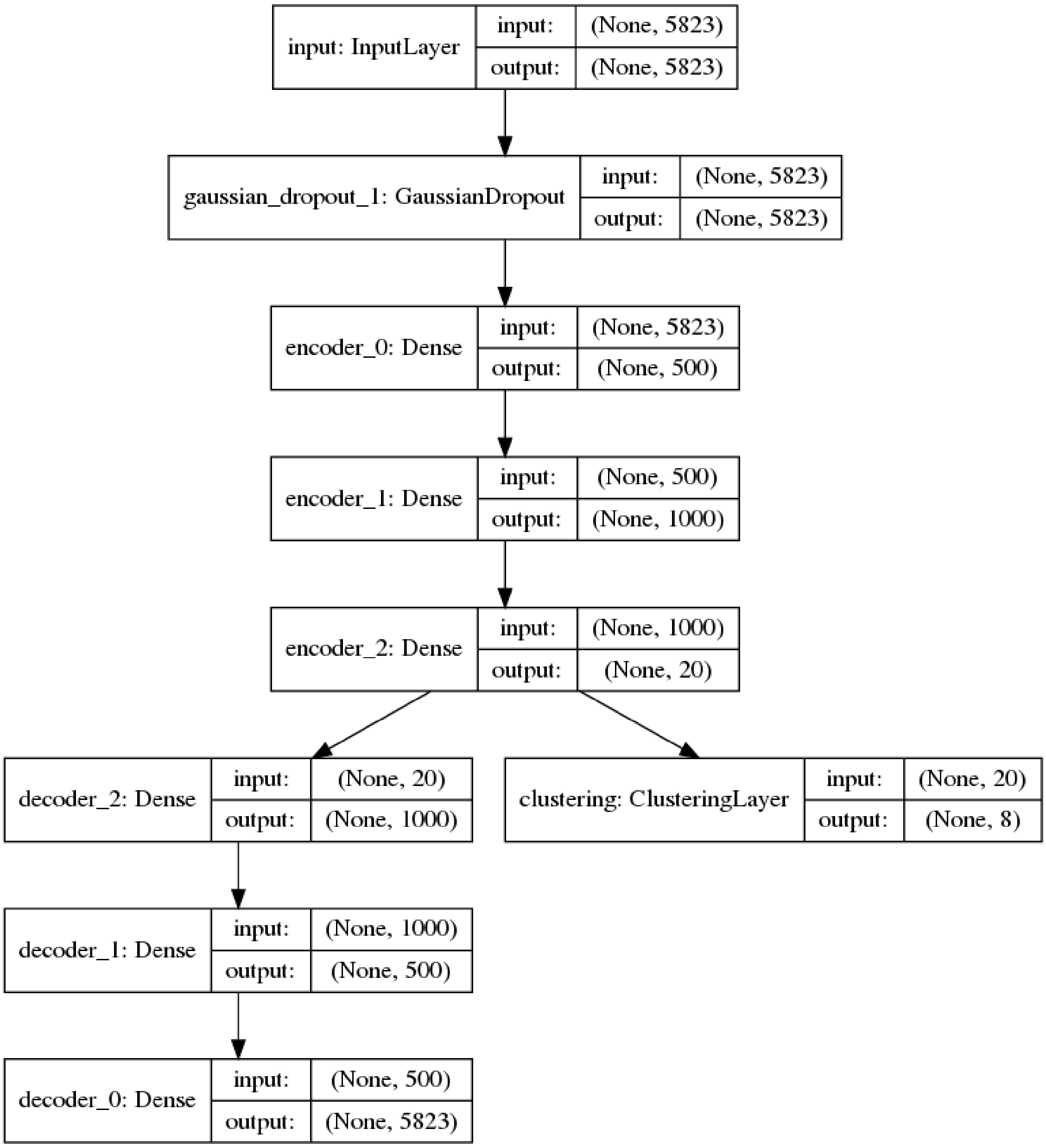

Figure 3: Architecture of deepAMR_cluster, a variant of deepAMR, where a deep denoising auto-encoder is augmented by a clustering layer at the most inner layer of the auto-encoder. The clustering layer is initialised by K-means clustering.

Supplement D

Table 4: Comparison between models on F2 feature sets.

Drugs \ Models		SVM	RF	MLKNN	ECC	DeepAMR
INH	Sen	72.2	72.7	58.8	65.7	**77.0**
	Spec	95.8	96.3	91.8	**98.4**	87.5
	AUROC	86.5	**87.1**	77.0	82.1	87.0
	F1	79.3	**80.1**	65.9	77.5	74.4
EMB	Sen	82.8	80.4	64.7	73.9	**85.3**
	Spec	92.4	93.1	**95.3**	94.7	92.2
	AUROC	89.7	91.7	84.6	84.9	**92.5**
	F1	70.9	71.1	65.9	70.8	**71.8**
RIF	Sen	80.1	78.0	58.1	74.3	**80.4**
	Spec	96.7	96.2	91.6	**96.8**	91.5
	AUROC	**90.9**	90.4	80.3	86.4	90.7
	F1	**83.4**	81.3	61.5	80.0	76.1
PZA	Sen	81.0	75.6	34.9	72.5	**82.7**
	Spec	92.1	94.9	**97.5**	94.0	89.6
	AUROC	89.1	88.3	81.2	84.3	**89.6**
	F1	67.5	**70.8**	45.5	66.8	63.5
MDR	Sen	81.1	79.9	64.8	75.8	**82.8**
	Spec	94.3	94.2	**95.5**	94.8	91.1
	AUROC	87.7	87.0	80.1	85.3	**91.7**
	F1	73.7	72.8	66.2	71.6	**76.0**
PANS	Sen	**78.9**	74.9	59.8	63.7	76.0
	Spec	93.7	95.5	88.9	**98.5**	87.5
	AUROC	86.3	85.2	74.4	81.1	**86.4**
	F1	**82.1**	81.3	65.4	76.3	75.2

Supplement E

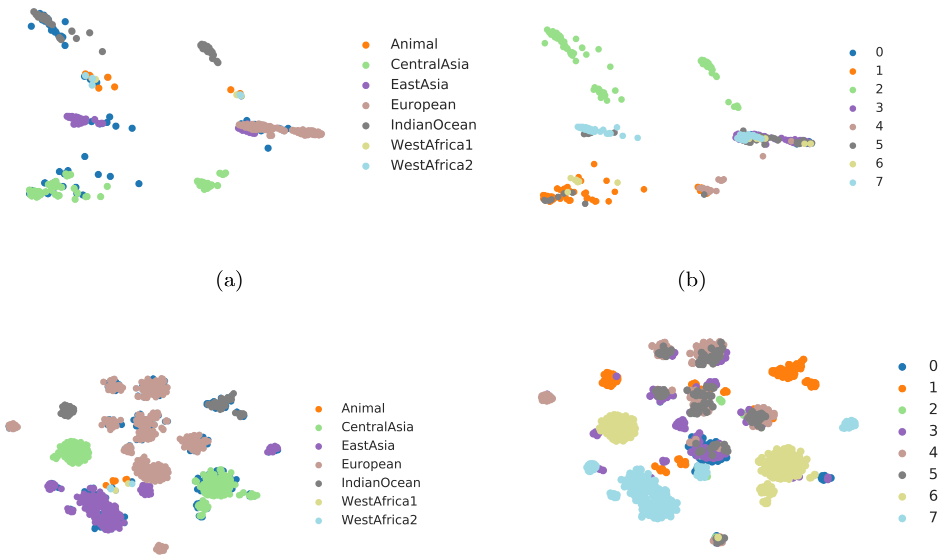

(a) (b)

(c) (d)

Figure 4: Visualization for the original input: a) lineage distribution using PCA; b) predicted clusters using PCA; c) lineage distribution using t-SNE; d) predicted clusters using t-SNE.

Supplement F

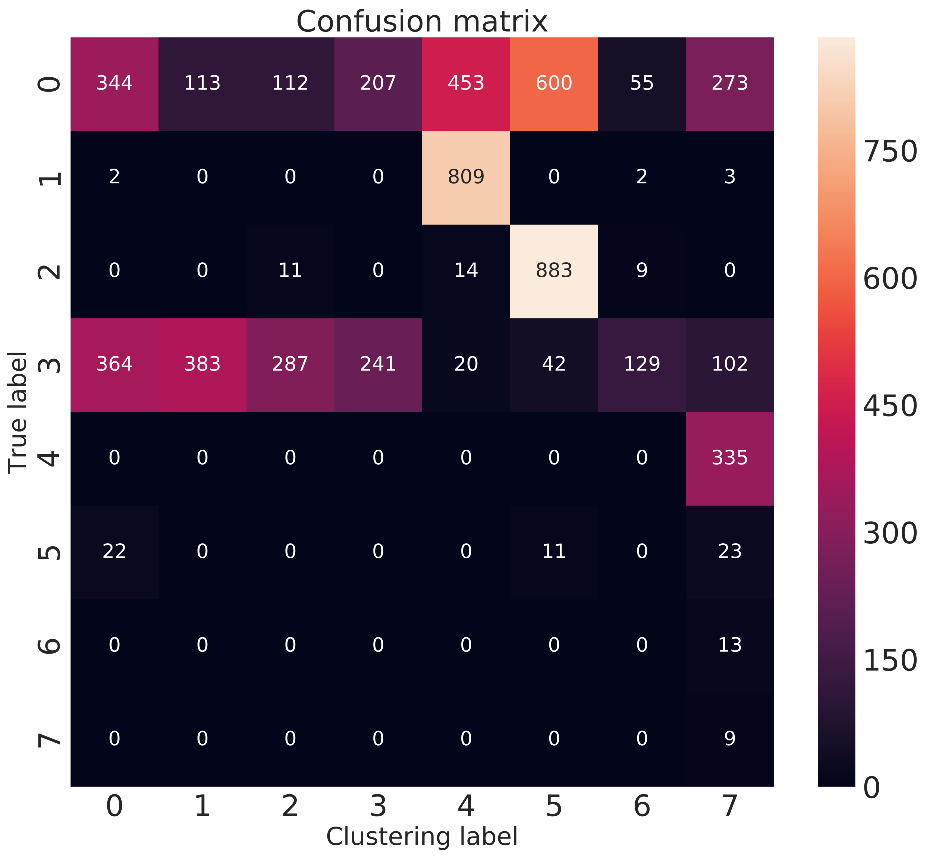

Figure 5: Heatmap of confusion matrix obtained by clustering.

Supplement H

Table 5: Hyperparameters tuning for examined machine learning methods.

Methods	Hyperparameter grid	Implementation	Internal CV
SVM	Linear Kernel, regularisation param: C=[0.001, 0.01,0.1,1,100]	scikit-learn	Yes
RF	N_estimatros=[10,20,30], max_features=[auto,sqrt,log2], min_samples_split=[2,4,8], Bootstrap=[True, False]	scikit-learn	Yes
MLKNN	Number of neighbours: k=[1,3,5], smoothing param: s=[0.5, 0.7, 1.0]	scikit-multilearn	Yes
ECC	Base leaner=logisticRegrassion, n_chains=20, order=random	scikit-learn	No
DeepAMR	batch size=64, kernel_initializer=uniform, drop_rate=0.3, deepAMR_optimizer=Nadam, learning rate=CLR(triangle2)[1], ae_optimizer=SGD	Keras	No
DeepAMR_cluster	batch size=64, kernel_initializer=uniform, drop_rate=0.3, cluster_initializer=K-means, N_cluster=8, ae_learning rate=CLR(triangle2) [1],ae_optimizer=SGD	Keras	No

Note: Internal CV means that the internal cross validation grid search is conducted in every experiment across over hyperparameter grid; ae is short for auto-encoder. All models were implemented by Python 3.6. Package requirements: numpy, pandas, tensorflow, keras, scipy, matplotlib, sklearn, seaborn,iterstrat, clr_callback

References

[1] Smith, Leslie N. *Cyclical learning rates for training neural networks.* Applications of Computer Vision (WACV), 2017 IEEE Winter Conference on, 464–472, 2017.

Supplement I

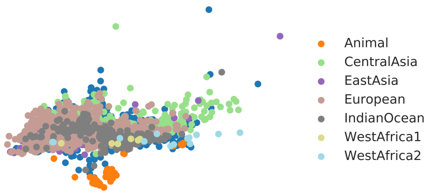

(a)

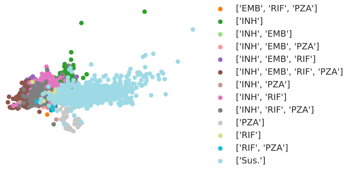

(b)

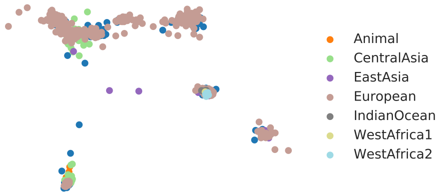

(c)

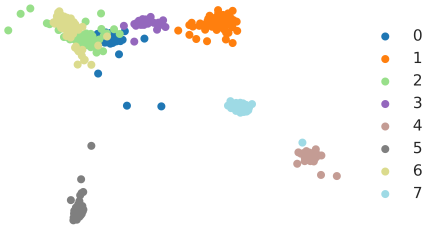

(d)

Figure 6: Visualization obtained by PCA: a) lineage distribution in latent space learned by DeepAMR; b) phenotype distribution in latent space learned by DeepAMR; c) lineage distribution in latent space learned by DeepAMR_cluster; d) predicted clusters in latent space learned by DeepAMR_cluster.
